# Risk Perception: It’s Personal

**DOI:** 10.1289/ehp.122-A276

**Published:** 2014-10-01

**Authors:** Valerie J. Brown

**Affiliations:** Valerie J. Brown, based in Oregon, has written for *EHP* since 1996. In 2009 she won a Society of Environmental Journalists’ Outstanding Explanatory Reporting award for her writing on epigenetics.

Risk perception is a highly personal process of decision making, based on an individual’s frame of reference developed over a lifetime, among many other factors. A body of research from the past several decades makes it clear that when it come to making decisions about health and safety, we don’t always worry the most about the most pressing threats.^1^^,^^2^ Risk consultant David Ropeik calls this the “risk perception gap.”

**Figure:**
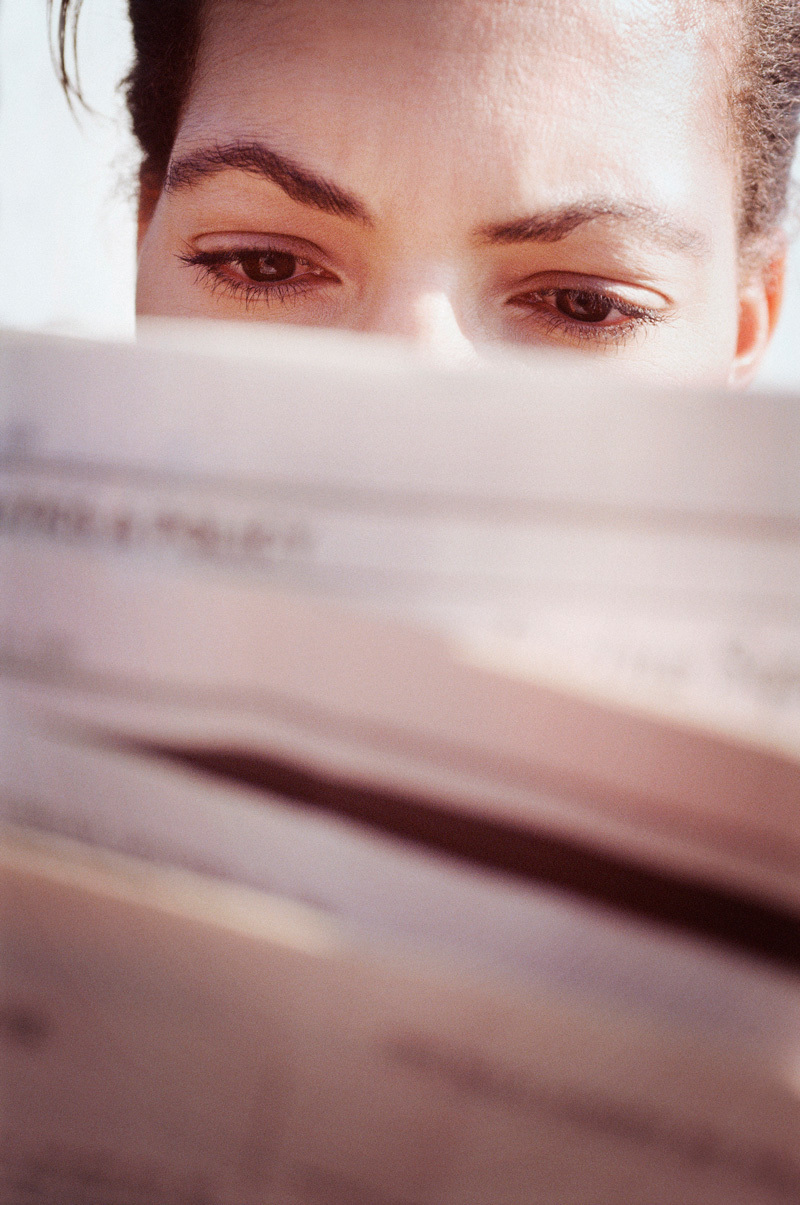
In the face of contradictory information, people must rely on their instincts as much as the facts to size up potential threats. © Corbis

On the surface, this risk perception gap may appear to be a result of ignorance. However, experts including Ropeik, University of Oregon psychologist Paul Slovic, and many more say that, in fact, it’s a natural extension of our hard-wired ability to quickly size up threats, an ability that draws on much more than facts alone. “The older view is that the public is emotional and hence irrational,” Slovic says. “But that’s not correct. Emotions are an extraordinarily sophisticated form of intelligence,” he says, “born out of millennia of quickly assessing high risks.”

## Thinking about Risk

Ropeik believes the difference between the way experts and the public think about risk sometimes creates risks all by itself. To a scientist who conducts risk assessments, the definition of risk is “hazard times exposure equals consequence,” he says. But to the average person, the definition of risk is “the probability of something bad happening.” And risk communication does not always account for the subjectivity of “something bad.”

In a regulatory or research setting, risk assessment typically entails a four-step process: hazard identification, hazard characterization, exposure assessment, and risk characterization.^3^ Both quantitative and qualitative expressions of risk, and some indication of uncertainties, are incorporated into the process. The goal is to arrive at a decision based on the most rational analysis of the best available evidence. Environmental health scientists are exploring new ways to strengthen the integrity of this process using principles of systematic review.^4^^,^^5^

Individuals mentally assess risk in a similar way, but risk perception is shaped by several largely unconscious emotional processes shared by scientists and nonscientists alike. For one, the human brain is hard-wired to react quickly and defensively to perceived threats of any kind.^6^ This includes physical threats, sights, sounds, smells, and even words or memories associated with fear or danger. For example, just the word “chemicals,” a common part of the environmental health lexicon, has been shown to trigger an unconscious fear reaction in members of the general public.^7^^,^^8^

Another largely unconscious process is the use of mental shortcuts to quickly make sense of partial information.^9^ One such shortcut is to map partial information against patterns of what we already know^9^—in a sense, judging a book by its cover. If our associations are negative, we will react fearfully, says Ropeik, “and if our associations are not negative, we might not react with as much caution as we should.”

Third, different characteristics of a threat carry different weights in terms of how people perceive the risk involved. For instance, threats that are uncontrollable, involuntary in nature, or cause a potential risk to future generations tend to cause more anxiety among the general public than threats that can be controlled or undertaken voluntarily.^1^ Finally, people tend to shape their views so they match those in the groups with which they most closely relate, a concept known as cultural cognition.^10^

## Challenges to Risk Communication

Effective risk communication depends on acknowledging the many factors that contribute to individual risk perception and aims to help people combine instinct with evidence to make the healthiest choices possible.

Of all the emotional aspects of risk communication, trust is perhaps the most pivotal. Scientists and other experts who routinely speak to lay groups about environmental health issues find that people will come to an issue with a great deal of fear, anger, and mistrust if they feel their concerns have already been mishandled. What makes people angriest and least trusting is when they either don’t know what the risks of an exposure are, feel they have been misled about the risks, or have been exposed without their consent, says Tracey Woodruff, a professor in the Department of Obstetrics, Gynecology, and Reproductive Sciences at the University of California, San Francisco.

Crisis communicators ran into these problems in Charleston, West Virginia, following the January 2014 spill of the industrial chemical crude MCHM into the Elk River. The spill contaminated the drinking water of some 300,000 people,^11^ and for days health officials had few firm facts to share with angry, alarmed residents.^12^ When the crisis was finally over, Rahul Gupta, executive director of the Kanawha–Charleston Health Department, noted that what worked best to establish trust was for officials to be frank about the limits of their knowledge and tell townspeople when they didn’t have answers.^12^

There is also a challenge with what is known as innumeracy, the struggle many people have understanding numbers, particularly probabilities.^13^ Even so, it’s often surprising how well nonexperts can handle probabilities and uncertainties, says Woodruff. “They have a good nuanced understanding,” she says. “You can tell them we might not know too much about the health risks, and they know how to conceptualize that.”

Some communicators find members of the public are savvier about environmental health than they used to be. Sharyle Patton is director of the Biomonitoring Resource Center for Commonweal, a nonprofit health and environmental research institute in Bolinas, California. She often brings in scientists to speak to community groups concerned about local environmental exposures. “Compared to ten years ago when we first started doing this work, lots of people [now] already know what ‘body burden’ is,” says Patton. As they learn more, she says, people “tend to want more information because they get really interested, and the more information they have, the less scared they are.”

Pat Hunt, a geneticist in Washington State University’s School of Molecular Biosciences in Pullman, often talks to the public about her work, which includes studying potential reproductive effects of endocrine-disrupting chemicals such as bisphenol A. She says, “I find that people are really responsive. They want to know, they want to be informed consumers.”

## At the Societal Level

But simply giving people lists of individual actions to mitigate risk isn’t enough. And Rachel Morello-Frosch, a professor in the School of Public Health at the University of California, Berkeley, says it misplaces the burden to expect individuals to do their own risk assessment.

“I think the assumption here is we’re expected to do our own risk assessment with everything,” says Morello-Frosch. “You cannot shop every day and do your personal risk assessment when you’re making [these] decisions.” She adds, “I think emotions around risk also emerge from very legitimate views on the extent to which people have control over their ability to minimize risks.”

Bruce Lanphear, an epidemiologist at Simon Fraser University in Burnaby, British Columbia, agrees that trust and control are pivotal parts of the risk perception equation. For instance, he says, until recently,^14^ federal agencies failed to promulgate regulations to reduce ongoing mercury emissions. Instead, the burden of reducing methylmercury exposure was shifted onto the consumer, with complicated and sometimes conflicting advice about fish consumption^15^ that he says left people with little sense of trust or control.

Pessimism about altering one’s risk can result not only from the fact that many risks—such as mercury pollution—are imposed at the population level, but also out of political and economic powerlessness. As Slovic wrote in 1999, “Whoever controls the definition of risk controls the rational solution to the problem at hand. … Defining risk is thus an exercise in power.”^16^

## Precaution

Carolyn Williams, technical director at the Institute of Risk Management in London points to the difficulties of making risk decisions when the science is not yet clear. The Institute of Risk Management teaches its students to gather the most reliable information and consult experts before making risk decisions. “We try to teach our students an approach to risk that helps organisations navigate a course between the ‘do nothing unless it’s proved 100% safe’ and the ‘do anything with no regard for safety’ extremes,” she says. Still, says Williams, “You’re going to have difficulties at the limit of scientific knowledge” where you have to rely on the intuition of experts.

Sometimes that intuition leads researchers to invoke the Precautionary Principle, to wit, when an activity raises threats of harm to human health or the environment, precautionary measures should be taken even if causal relationships have not been fully established.^17^ Critics have called it the Paralyzing Principle,^18^ but proponents maintain that precaution is reasonable when it is based on reliable information. As Ropeik puts it, “When we don’t have the facts, we rely on our *sense* of potential danger to protect us.”

If this is true, then in risk calculation might it be necessary to accord some weight to intuition or the sense that “something bad might happen”? “There’s a wisdom in feelings that we have to accept,” says Slovic. The challenge, then, is not so much to eliminate emotion as to harness its power without distorting the scientific evidence.
